# Supplementary material for: The Nucleosome Remodelling and Deacetylation complex suppresses transcriptional noise during lineage commitment
Source: EMBO J. 2019 Apr 29;38(12):e100788. doi: 10.15252/embj.2018100788 (PMC6576150; doi:10.15252/embj.2018100788)
Supplement: Supplementary file 1 — Appendix [file EMBJ-38-e100788-s001.pdf]

**Appendix Figure 1. High correlation amongst replicates of ChIP-seq data**

**Appendix Figure 2. *Mta3* stop codon selection in ES cells**

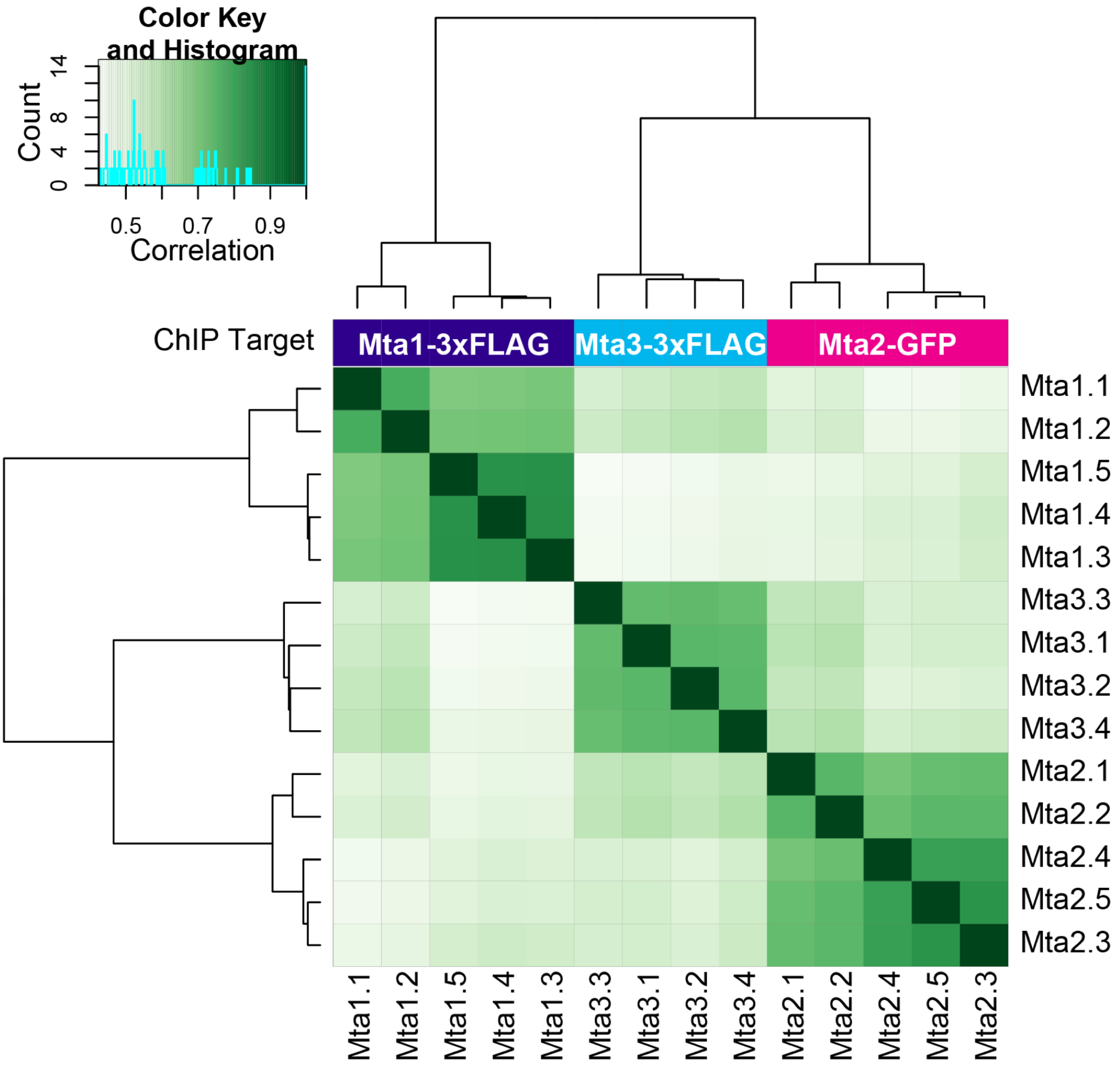


**Appendix Figure S1. High correlation amongst replicates of ChIP-seq data**

A genome-wide ChiP-seq signal correlation plot displaying all replicates included in the MTA ChIP-seq data. Though Mta3-3xFLAG showed lower overall signal, the degree of correlation amongst replicates is high.

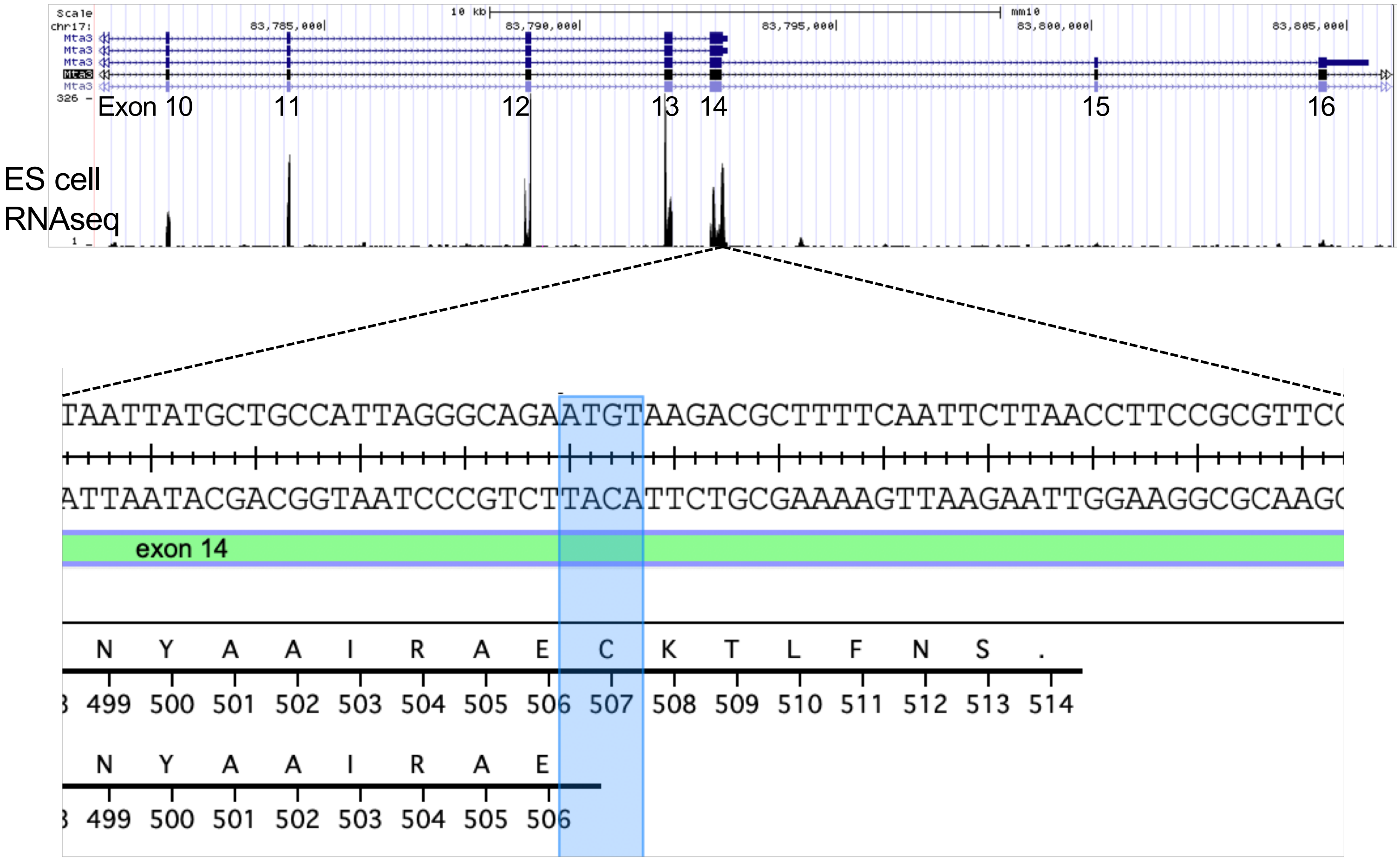


**Appendix Figure S2. *Mta3* stop codon selection in ES cells**
Screenshot from the UCSC Genome Browser including exons 10-16 of the *Mta3* locus (Top) and a wild type ES cell RNAseq trace. The sequence of a portion of exon 14 is shown below, with the amino acid sequence of alternate transcripts indicated and the wild type sequence of the splice donor mutated in the Mta3-Avi-3xFLAG allele highlighted.
