# Supplementary material for: The Nucleosome Remodelling and Deacetylation complex suppresses transcriptional noise during lineage commitment
Source: EMBO J. 2019 Apr 29;38(12):e100788. doi: 10.15252/embj.2018100788 (PMC6576150; doi:10.15252/embj.2018100788)
Supplement: Supplementary file 2 — Expanded View Figures PDF [file EMBJ-38-e100788-s002.pdf]

Expanded View Figures

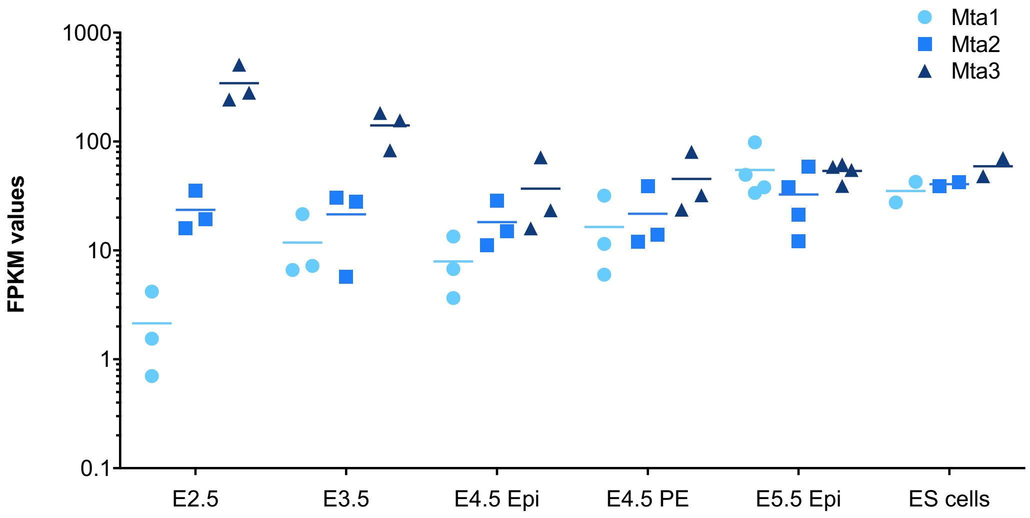

Figure EV1. Expression of *MTA* genes during early mouse development.
RNA-seq data from Boroviak *et al* (2014) plotted at indicated days of mouse development for each of the *MTA* genes. All data points are shown, with horizontal bars indicating the mean.

Figure EV2. Chromatin features of MTA-bound peaks.

A Comparison of ChIP-seq peaks for Mta3 and Chd4. The number of Mta3-only, Chd4-only or Mta3 + Chd4 peaks is indicated.
B Features of peaks containing two of the three MTA proteins, as in Fig 2D.
C Distribution of ChIP-seq peaks identified as shared amongst all three MTA proteins (MTA123) or as unique for indicated MTA proteins is plotted relative to Refseq transcription start sites (TSS) on the *x*-axis. Note distance is on a $\log_{10}$ scale.
D Average enrichment of indicated features is shown for peaks for each MTA protein which do or do not also contain Chd4. The number of peaks in each set is indicated as *n*.
E Average enrichment of DNA methylation across MTA peaks with or without Chd4, as in Panel (D).
F Significance of GO term enrichment for genes associated with peaks for different combinations of MTA proteins with or without Chd4.

▶

© 2019 The Authors

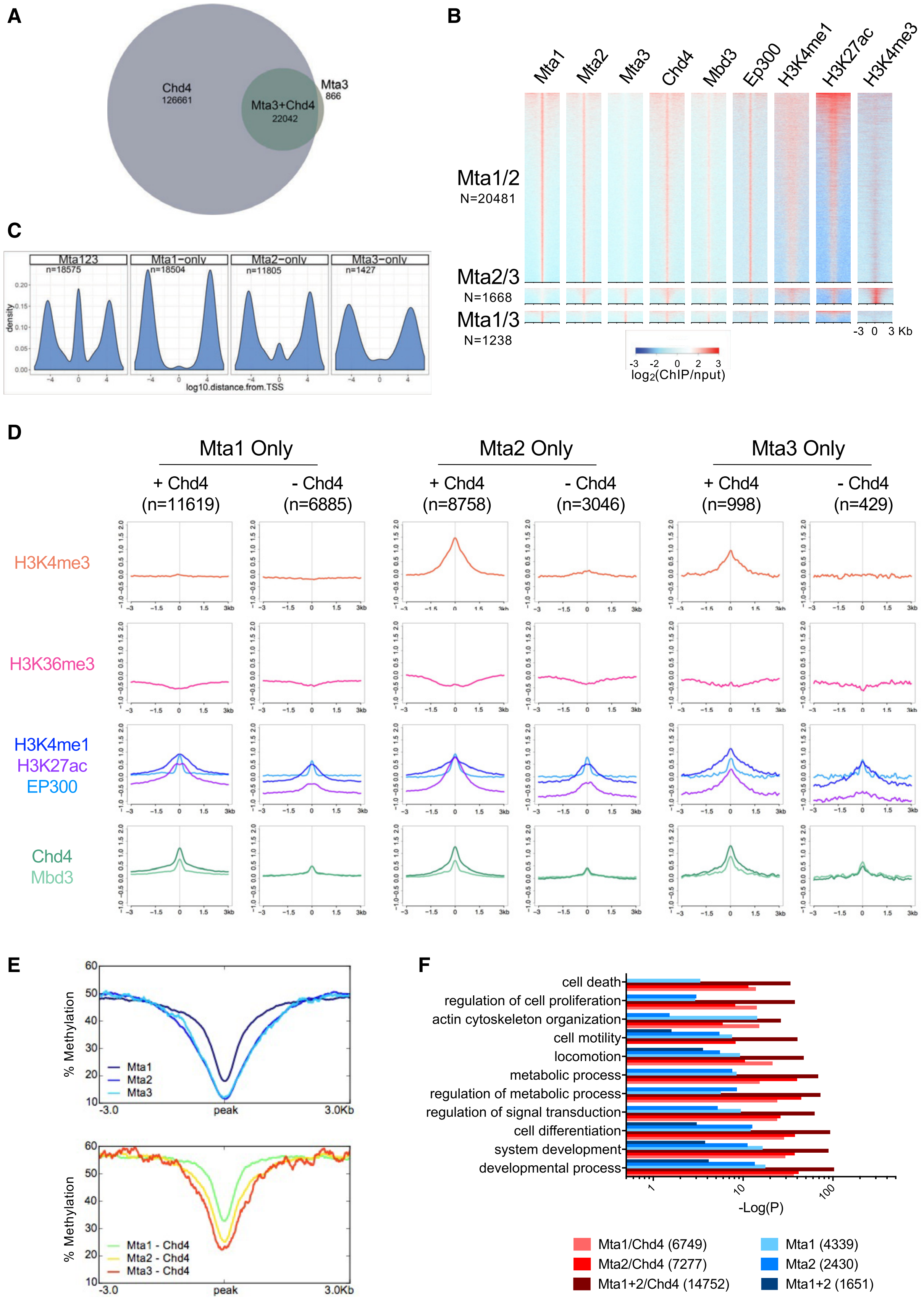

Figure EV2.

© 2019 The Authors

Figure EV3. *Mta* reporter and knockout alleles.

A Schematic of the *Mta1* "Knockout First" reporter allele *Mta1*$^{tm1a(EUCOMM)Wtsi}$ (Top). Exons are depicted as boxes, with normal coding exons as filled boxes. Exons around the insertion site are numbered. Coding exons not able to be translated in the depicted allele are shaded in light blue. The targeting resulted in an FRT-flanked LacZ-Neo fusion protein being expressed from the endogenous *Mta1* promoter and preventing transcription of most *Mta1* exons. Recombination between FRT sites is achieved by expression of FLP recombinase (Middle), removing the LacZ-Neo cassette and restoration of Mta1 coding potential. Subsequent recombination between LoxP sites by Cre recombinase (Bottom) results in loss of exon 2 and subsequent exons are out of frame.

B Schematic of the *Mta2*$^{tm1a(EUCOMM)Wtsi}$ allele as in panel (A). In this allele, the neo gene is expressed from a human ß-actin promoter and expression of Cre results in excision of exons 4–13.

C Schematic of the *Mta3*$^{tm3a(KOMP)Wtsi}$ allele as in panel (A).

D Western blot of nuclear extracts made from each single mutant probed with indicated antibodies. Anti-Rbp1 NTD (RNA polymerase II) acts as a loading control.

E Phase-contrast images of cell lines of indicated genotype in self-renewing conditions. Scale bars indicate 100 μm.

F Comparison of gene expression in indicated *Mta* single mutant ES cells and wild-type (WT) ES cells. Each circle represents a gene: red indicates genes that are not differentially expressed to a significant degree, and green indicates differentially expressed genes. The number of up- and downregulated genes defined with an adjusted P-value < 0.05, and a log2 fold-change > 1 is indicated in each plot. $N = 3$ for each genotype.

G Overlap of misexpressed genes in indicated single mutant ES cells. The number of genes in each overlap category is indicated.

H Gene expression data for each single *Mta* mutant ES line, the *Mbd3*Δ ES line and the *Mta123*Δ ES line and their control parental lines (WT) in self-renewing (2iL) conditions are plotted relative to that of mouse embryonic single cell RNA-seq data from Mohammed *et al* (2017) (smaller grey circles).

© 2019 The Authors

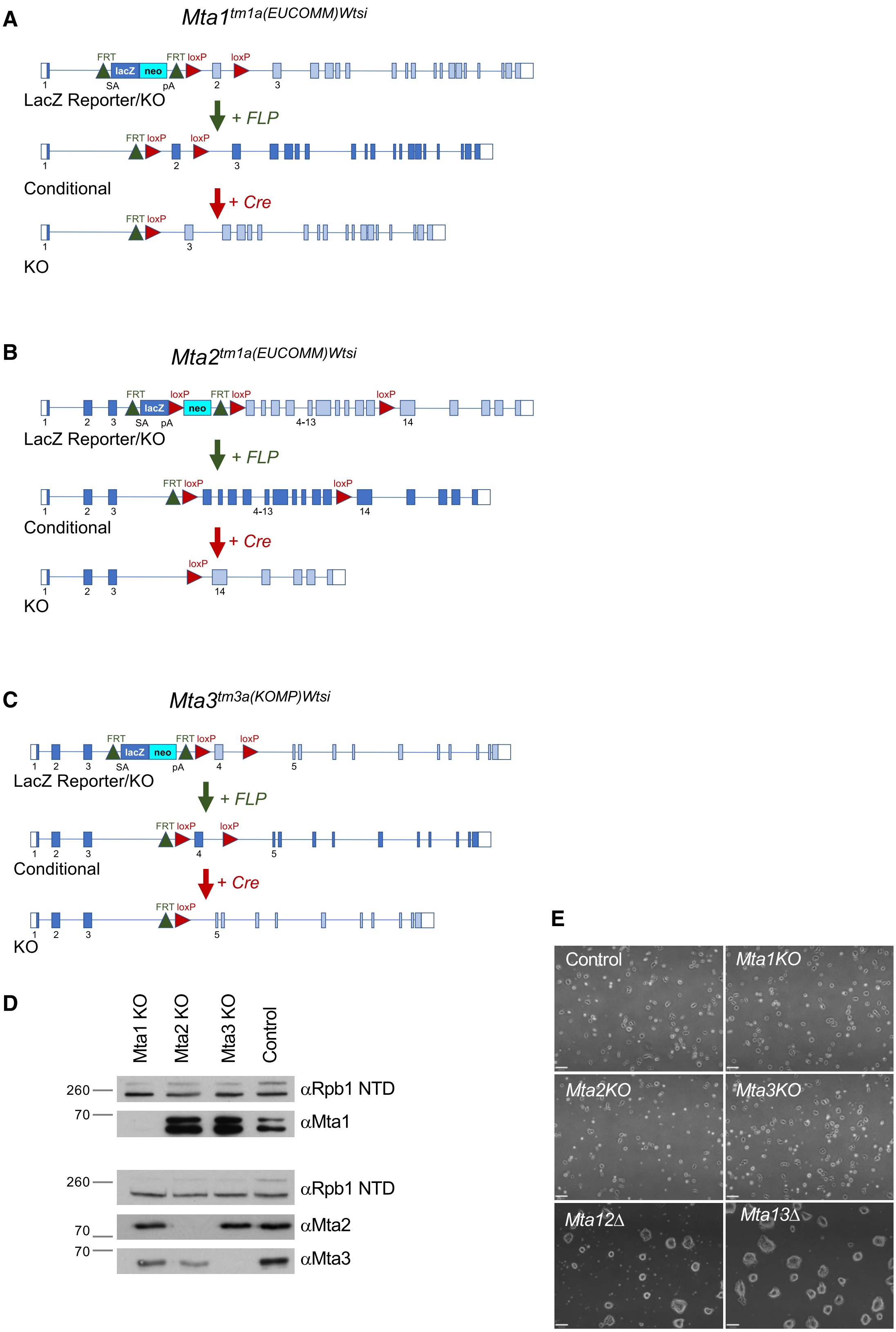

Figure EV3.

 © 2019 The Authors

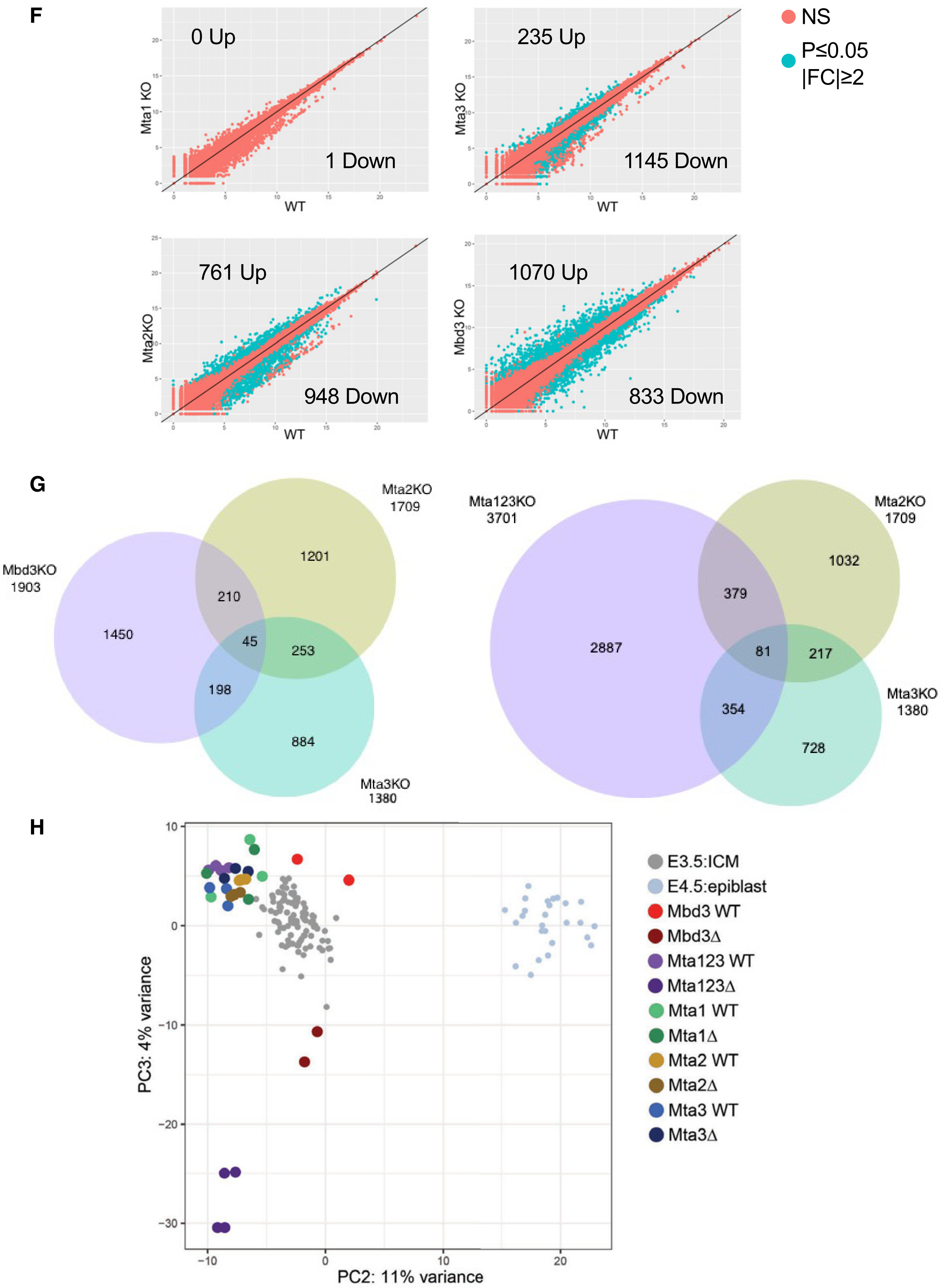

Figure EV3 continued.

Figure EV4. Control of gene expression by the MTA proteins.

A Western blot of nuclear extract from cell lines indicated at the top in 2iL conditions probed with antibodies listed at right. Arrows indicate the locations of the two Mbd2 isoforms in the anti-Mbd2 panel, and of the three Mbd3 isoforms in the anti-Mbd3 panel. The grey triangle indicates the location of Mbd3-3xFLAG, which was present in the *Mbd2*Δ line used. A non-specific band at ~ 40kDa is present in all lanes in the αMbd2 panel. Anti-Rbp1 NTD (αRNAPII) acts as a loading control. Approximate sizes are indicated at left in kDa.

B Western blot of indicated proteins in wild-type and *Mbd3*Δ ES cells in 2iL conditions. Anti-Rbp1 NTD (αRNAPII) acts as a loading control.

C Comparison of gene expression changes in wild-type (*Mta123WT* = control ES cell line derived at the same time as the parent of the *Mta123*Δ ES cell line), *Mbd3*Δ and *Mta123*Δ ES cells in self-renewing (2iL) conditions. The top 100 genes contributing to PC2 from Fig 5E are shown.

D Common ChIP-seq peaks for Chd4 and all three MTA proteins (Core NuRD peaks) were assigned to the nearest genes. These genes were then divided into four equal groups based upon expression levels in wild-type cells, and their expression in *Mbd3*Δ ES cells (top panels) or *Mta123*Δ ES cells (bottom panels) is plotted relative to that in wild-type ES cells. Change in gene expression is plotted on the *x*-axis and calculated significance of the expression change on the *y*-axis. Those genes significantly misexpressed ($|\log2FC| > 1$; $P \leq 0.05$) are indicated in red, and genes showing no significant change are plotted in black. The percentage of misexpressed genes located within each quartile is indicated above each graph.

© 2019 The Authors

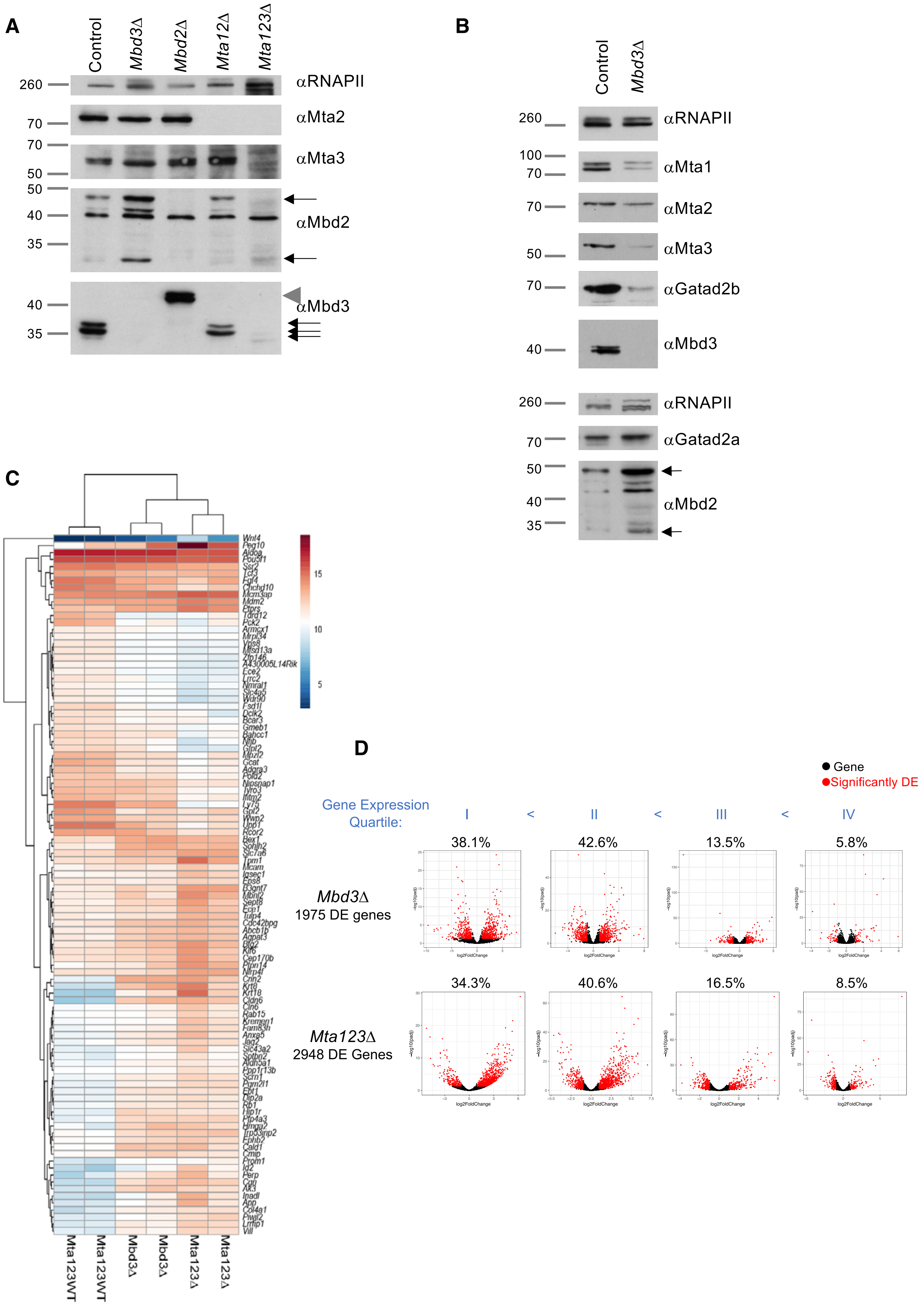
A
Control
Mbd3Δ
Mbd2Δ
Mta12Δ
Mta123Δ
260
αRNAPII
70
αMta2
70
50
αMta3
50
40
35
αMbd2
40
35
αMbd3
B
Control
Mbd3Δ
260
αRNAPII
100
70
αMta1
70
αMta2
50
αMta3
70
αGatad2b
40
αMbd3
260
αRNAPII
70
αGatad2a
50
40
35
αMbd2
C
15
10
5
Mta123WT
Mta123WT
Mbd3Δ
Mbd3Δ
Mta123Δ
Mta123Δ
D
Gene
Significantly DE
Gene Expression Quartile:
I < II < III < IV
38.1%
42.6%
13.5%
5.8%
Mbd3Δ
1975 DE genes
34.3%
40.6%
16.5%
8.5%
Mta123Δ
2948 DE Genes

Figure EV4.

Figure EV5. Failure of differentiation in *Mta123*Δ ES cells.

A Phase-contrast images of ES cells of indicated genotypes induced to differentiate for 5 days in N2B27. Scale bars represent 100 μm.
B Comparison of gene expression in indicated ES cell lines in undifferentiated conditions or after 5 days in neuroectoderm differentiation conditions (N2B27). RT–qPCR was carried out in triplicate at each time point for a minimum of three biological replicates. Error bars indicate SEM.
C GO term enrichment for genes differentially expressed in *Mbd3*Δ ES cells (left) or *Mta123*Δ ES cells (right) after 48 h of differentiation in N2B27. For each comparison, the top five most significant gene ontology terms are plotted by $\log_{10}$ of the Benjamini-adjusted *P*-value. Significant GO terms and *P*-values were calculated using David v.6.8 (da Huang *et al*, 2009a).
D Expression of genes associated with indicated GO terms is plotted by fold-change in expression in *Mta123*Δ ES cells (*x*-axis) or *Mbd3*Δ ES cells (*y*-axis) after 48 h of differentiation in N2B27. Genes are coloured if they are differentially expressed (log2 fold-change > 1 and P_{adj} value < 0.05) in either comparison as indicated. The dotted lines show the fold-change cut-off of 2. GO terms were identified using David v.6.8 using a Benjamini score with a cut-off of 0.05.
E Comparison of gene expression in indicated ES cell lines induced to differentiate towards mesoderm. qPCR was carried out in triplicate at each time point for a minimum of three biological replicates. Error bars indicate SEM.
F Same as Fig 5E, plotting PC4 vs. PC1.
G Loading plot for Fig 5E. A selection of key genes is highlighted in different colours based upon a published classification of early embryonic gene expression (Boroviak *et al*, 2014).
H Composite images of representative chimaeric embryos as in Fig 6A stained with an anti-activated Caspase-3 antibody. Arrows indicate an example of an orange, apoptotic cell. Scale bars = 20 μm.

 © 2019 The Authors

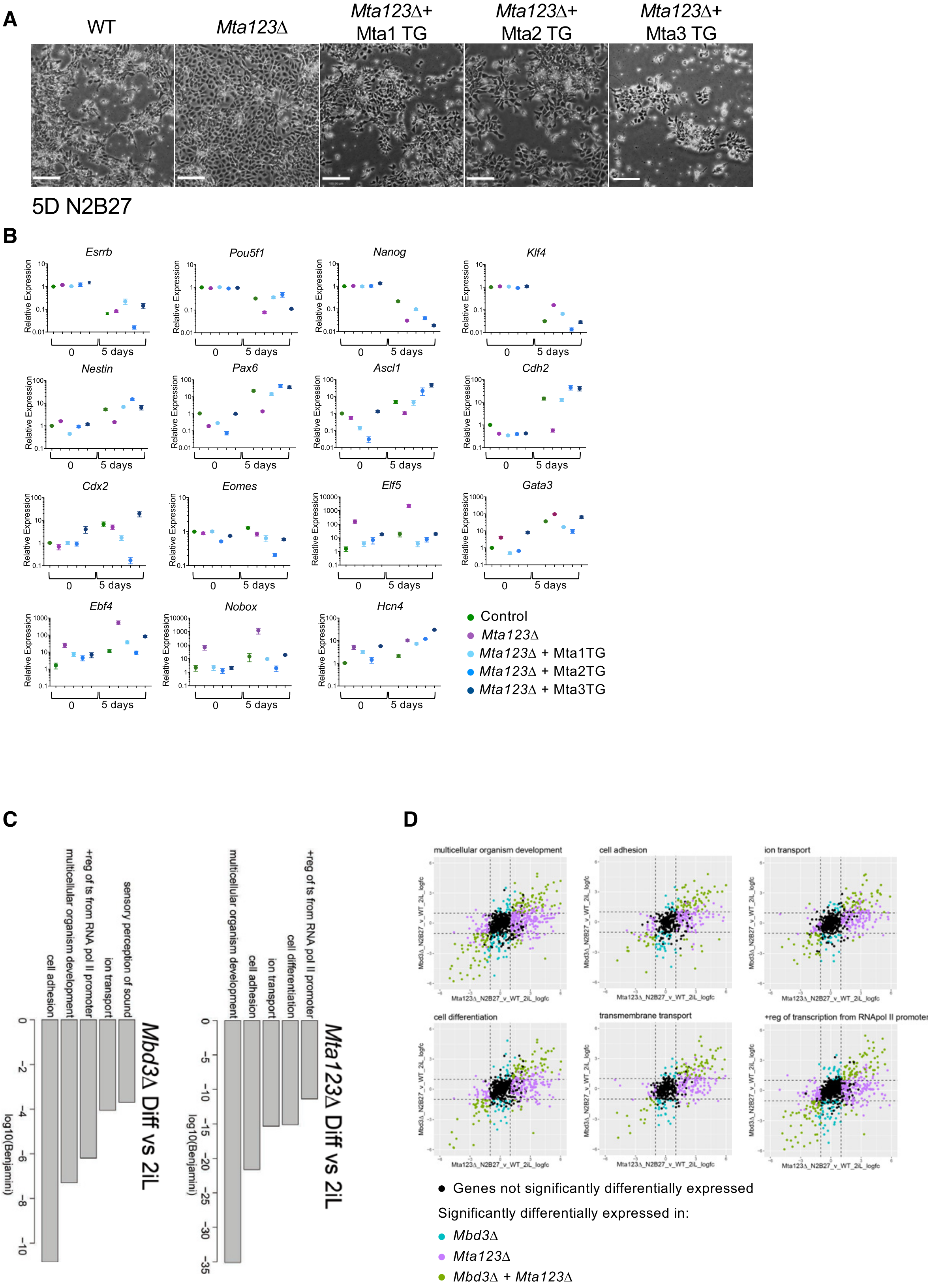
A
WT
Mta123Δ
Mta123Δ+ Mta1 TG
Mta123Δ+ Mta2 TG
Mta123Δ+ Mta3 TG
5D N2B27
B
Esrrb
Pou5f1
Nanog
Klf4
Nestin
Pax6
Ascl1
Cdh2
Cdx2
Eomes
Elf5
Gata3
Ebf4
Nobox
Hcn4
Relative Expression
0
5 days
Control
Mta123Δ
Mta123Δ + Mta1TG
Mta123Δ + Mta2TG
Mta123Δ + Mta3TG
C
Mbd3Δ Diff vs 2iL
cell adhesion
multicellular organism development
+reg of ts from RNA pol II promoter
ion transport
sensory perception of sound
log10(Benjamini)
Mta123Δ Diff vs 2iL
multicellular organism development
cell adhesion
ion transport
cell differentiation
+reg of ts from RNA pol II promoter
D
multicellular organism development
cell adhesion
ion transport
cell differentiation
transmembrane transport
+reg of transcription from RNApol II promoter
Mbd3Δ_N2B27_v_WT_2iL_logfc
Mta123Δ_N2B27_v_WT_2iL_logfc
Genes not significantly differentially expressed
Significantly differentially expressed in:
Mbd3Δ
Mta123Δ
Mbd3Δ + Mta123Δ

Figure EV5.

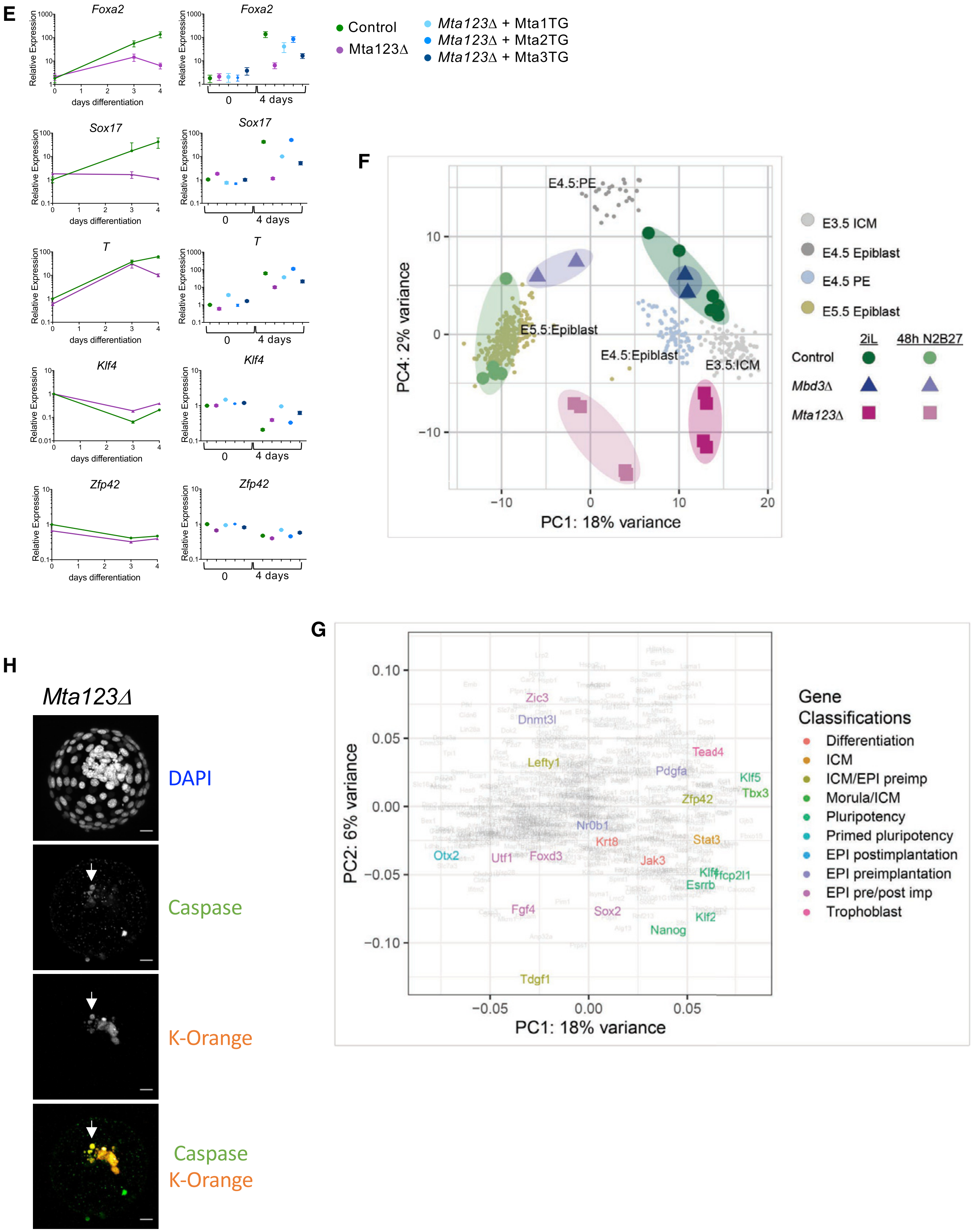

Figure EV5 continued.

 © 2019 The Authors
