# Supplementary material for: The Nucleosome Remodelling and Deacetylation complex suppresses transcriptional noise during lineage commitment
Source: EMBO J. 2019 Apr 29;38(12):e100788. doi: 10.15252/embj.2018100788 (PMC6576150; doi:10.15252/embj.2018100788)
Supplement: Supplementary file 4 — Table EV2 [file EMBJ-38-e100788-s004.docx]

**Table EV2. Antibodies used in this study**

| **Antibody** | **Company**  **(Product Number)** | **WB** | **IP** | **IF** | **ChIP** | **RRID** |
| --- | --- | --- | --- | --- | --- | --- |
| anti-CASPASE-3 cleaved | Cell Signalling  (9664S) | - | - | 1:500 | - | RRID:AB_2070042 |
| anti-CDX2 | Abcam  (ab157524) | - | - | 1:250 | - | RRID:AB_2721036 |
| anti-CHD4 | Abcam  (ab70469) | 1:5000 | 5 μg | - | - | RRID:AB_2229454 |
| anti-FLAG M2 | Sigma Aldrich  (F1804) | 1:2000 | 3 μg | - | 10 μg | RRID:AB_262044 |
| anti-GATAD2A | Abcam  (ab87663) | 1:2000 | - | - | - | RRID:AB_1952305 |
| anti-GATAD2B | Bethyl Laboratories  (A301-281A) | 1:2000 | - | - | - | RRID:AB_937934 |
| anti-GFP | Abcam  (ab290) | 1:5000 | 15µg |  | 25µg | RRID:AB_303395 |
| anti-HDAC1 | Millipore  (06-720) | 1:2000 | - | - | - | RRID:AB_305705 |
| anti-HDAC2 | Santa cruz  (sc-7899) | 1:1000 | - | - | - | RRID:AB_2118563 |
| anti-Lamin B1 | Abcam  (ab133741) | 1:10000 | - | - | - | RRID:AB_2616597 |
| anti-MBD2 | Abcam  (ab58241) | 1:500 | - | - | - | RRID:AB_944120 |
| anti-MBD3 | Abcam  (ab157464) | 1:5000 | - | - | - |  |
| anti-MTA1 | Cell Signalling  (5647) | 1:2000 | - | - | - | RRID:AB_10705601 |
| anti-MTA2 | Abcam  (ab50209) | 1:5000 | 3 μg | - | - | RRID:AB_2146939 |
| anti-MTA3 | Proteintech  (14682-1-AP) | 1:2000 | - | - | - | RRID:AB_2298003 |
| anti-RBBP4 | Abcam  (ab79416) | 1:5000 | - | - | - | RRID:AB_1603754 |
| anti-RNA Pol II, RPB1 subunit | Cell Signalling  (14958S) | 1:5000 | - | - | - | RRID:AB_2687876 |
| anti-SOX2 | e-biosciences  (14-9811-82) | - | - | 1:500 | - | RRID:AB_11219471 |
